# Predictors of ICU Mortality among Mechanically Ventilated Patients: An Inception Cohort Study from a Tertiary Care Center in Addis Ababa, Ethiopia

**DOI:** 10.1155/2022/7797328

**Published:** 2022-12-08

**Authors:** Finot Debebe, Alberto Goffi, Tewodros Haile, Fetiya Alferid, Haimanot Estifanos, Neill K. J. Adhikari

**Affiliations:** ^1^Addis Ababa University, Addis Ababa, Ethiopia; ^2^Interdepartmental Division of Critical Care Medicine, University of Toronto, Toronto, Canada; ^3^Critical Care Department, Unity Health Toronto, Toronto, Canada; ^4^Saint Paul Millennium Medical College, Addis Ababa, Ethiopia; ^5^Department of Critical Care Medicine, Sunnybrook Health Sciences Centre, Toronto, Canada

## Abstract

**Background:**

Mechanical ventilation is a life-saving intervention for patients with critical illnesses, yet it is associated with higher mortality in resource-constrained settings. This study intended to determine factors associated with the mortality of mechanically ventilated adult intensive care unit (ICU) patients.

**Methods:**

A one-year retrospective inception cohort study was conducted using manual chart review in ICU patients (age >13) admitted to Tikur Anbessa Specialized Hospital (Addis Ababa, Ethiopia) from September 2019 to September 2020; mechanically ventilated patients were followed to hospital discharge. Demographic, clinical, and outcome data were collected; logistic regression was used to determine mortality predictors in the ICU.

**Result:**

A total of 160 patients were included; 85/160 (53.1%) were females and the mean (SD) age was 38.9 (16.2) years. The commonest indication for ICU admission was a respiratory problem (*n* = 97/160, 60.7%). ICU and hospital mortality were 60.7% (*n* = 97/160) and 63.1% (*n* = 101/160), respectively. Coma (Glasgow Coma Score <8 or 7 with an endotracheal tube (7T)) (adjusted odds ratio [AOR] 6.3, 95% confidence interval 1.19–33.00), cardiovascular diagnosis (AOR 5.05 [1.80–14.15]), and a very low serum albumin level (<2 g/dl) (AOR 4.9 [1.73–13.93]) were independent predictors of mortality (*P* < 0.05). The most commonly observed complication was ICU acquired infection (*n* = 48, 30%).

**Conclusions:**

ICU mortality in ventilated patients is high. Coma, a very low serum albumin level (<2 g/dl), and cardiovascular diagnosis were independent predictors of mortality. A multifaceted approach focused on developing and implementing context appropriate guidelines and improving skilled healthcare worker availability may prove effective in reducing mortality.

## 1. Introduction

Critical care is a well-established and essential component of the continuum of care in the developed world [1]. However, it has received little attention in the developing world [2–6]. Mechanical ventilation is one of the most widely used life-saving interventions in the intensive care unit (ICU) [7–11], but it is associated with complications [7, 11, 12].

Compared to high-resource settings, patients receiving mechanical ventilation in low-income settings have higher death rates [4, 6, 7, 9, 10, 13], which could be related to individual patient factors [9] or systemic factors. Patients in low-resource settings may be less likely to seek timely care due to a variety of reasons, including lack of awareness, access [4], and financial constraints [1]. They are more likely to be referred to the ICU with advanced illness [1, 9]. In addition, ICUs are few and inadequately equipped [3, 5]. For example, the Ethiopian nationwide ICU survey revealed a significant shortage of mechanical ventilators, with only 203 ventilators for 114 million people in the public sector [3]. This equipment is mostly used, donated, and infrequently maintained [4]. The problem is further intensified by the paucity of trained professionals [1–4].

For these reasons, the available scarce resources should be used effectively, [2, 5] and identification of factors related to patient mortality [9] may help to prioritize care for the most vulnerable patients. Although a larger observational study ascertained factors associated with the mortality of mechanically ventilated patients in ten middle-income Asian countries [12], data are lacking for Africa [14]. Therefore, the study primarily aimed to determine factors predicting the mortality of mechanically ventilated patients in the medical and surgical intensive care units of Tikur Anbessa Specialized Hospital, Addis Ababa, Ethiopia. The study's secondary aims were to identify the most common ICU complications among mechanically ventilated patients.

## 2. Materials and Methods

### 2.1. Setting and Design

Tikur Anbessa Specialized Hospital is located in Addis Ababa, the capital city of Ethiopia. The hospital is the biggest teaching and tertiary referral center in the country. It has 12 adult medical and surgical ICU beds. Patients over the age of 13 are admitted to these ICU beds. The patient-to-nurse to ratio is one to one. Pulmonology and critical care specialists and residents care for medical ICU patients, while anesthesiology and critical care specialists and residents care for surgical ICU patients. Patients with medical conditions that require critical care are admitted to the medical ICU. Perioperative, trauma, and obstetric/gynecologic patients usually get admitted to the surgical ICU. These ICUs providedcare for patients without COVID-19 infection.

A retrospective cohort study was conducted by reviewing all available charts of mechanically ventilated patients, with inception from September 2019 to September 2020; patients were followed to hospital discharge. A pretested case report form was developed by extracting key variables from previously published surveys in resource constrained settings.

### 2.2. Data Collection

**Figure 1:**
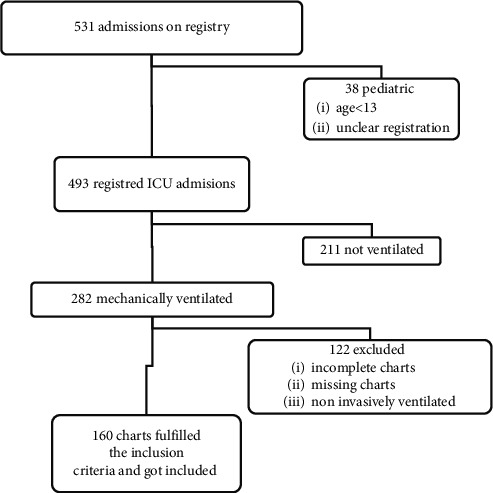
Flow diagram of patients admitted and mechanically ventilated in the intensive care unit of Tikur Anbessa Specialized Hospital, from September 2019 to September 2020, Addis Ababa, Ethiopia.

The ICU admission/discharge logbook was used as an entry point to identify participants (Figure 1). All charts containing at least 75% of data on the variables of interest and age greater than equal to 13 years old were included. Participants with missing/incomplete data (more than 25%) and those who were treated with noninvasive ventilation were excluded. Sociodemographic, clinical, and outcome data (mortality in the intensive care unit and at hospital discharge) were extracted.

Disease severity was assessed using the admission Mortality Prediction Model (MPM) II score [15] and ICU admission priority level, as classified using the Society of Critical Care Medicine categorization system [16], with the following four categories being used as follows:

- Priority 1: critically ill, receiving care that can only be provided in the ICU.
- Priority 2: patients needing intensive monitoring but not necessarily an ICU bed.
- Priority 3: acutely ill with a reduced chance of survival.
- Priority 4: irreversible disease condition with no difference in outcome due to ICU admission.

### 2.3. Statistical Analysis

Data were entered and analyzed using the Statistical Package for Social Sciences (SPSS) version 25. Continuous data were checked for normality and summarized as means with standard deviations (SDs) or medians with interquartile ranges (IQRs), whereas categorical data were summarized as frequencies and percentages. Variables were compared between survivors and nonsurvivors (at ICU discharge) using Pearson's chi-square tests for categorical variables, an independent sample *t*-test for continuous variables, and the Mann–Whitney *U* test for variables without normal distribution.

Backward stepwise multivariable logistic regression was conducted to identify independent predictors of mortality. All independent variables with *P* values less than 0.25 were initially considered to be included in the multivariable analysis. Using tolerance and the variance inflation factor, the selected variables were checked for multicollinearity. The model that best classified the outcome variables with a significant omnibus test and Hosmer and Lemeshow's goodness of fit test (*P* > 0.05) was selected as the final model. The results are presented as crude odds ratio and adjusted odds ratio (AOR) with 95% confidence interval and *P* values. To assess the robustness of the association between covariates related with ICU mortality for unmeasured confounding, sensitivity analysis using the E-value was performed [17].

## 3. Results

### 3.1. Baseline Characteristics

A total of 282 patients were mechanically ventilated, from which 160 charts fulfilling the inclusion criteria got enrolled. From these, 85 (53.1%) were female. The mean (SD) age was 38.9 (16.2) years. One hundred forty-five (90.6%) were <60 years old. Ten patients (6.3%) were admitted after cardiopulmonary resuscitation (CPR). The most common reason for ICU admission was a respiratory problem (*n* = 97/160, 60.7%). Thirty-five patients had a Glasgow Coma Scale (GCS) less than eight or seven with an endotracheal tube (7T) at admission to the ICU Table 1.

The mean (SD) admission Mortality Prediction Model (MPM) II score was 36.9 (25.5). The largest category (*n* = 77/160, 48.1%) comprised priority one admissions (critically ill patients requiring treatment and monitoring that cannot be delivered outside of ICUs). Only 48 patients had a documented serum albumin level, which was low in 45 (98%) patients (Table 1). Eighty-five percent of the participants (*n* = 136/160) had at least one comorbidity, notably, immunosuppression (*n* = 90, 56.3%) and hypertension (*n* = 32, 20%).

Respiratory failure (*n* = 72/160, 45%) was the most common reason for intubation and mechanical ventilation. The most frequently used initial mode of ventilation was assist control/volume control (AC/VC) mode (*n* = 88, 54.4%) Table 1.

The median (IQR) duration of mechanical ventilation was 3 (2–7) days. Only five patients underwent tracheostomy. Overall, seventy-five patients (46.9%) required vasopressor therapy during their stay in the ICU, while (*n* = 40, 25%) received blood transfusions. Adrenaline was the most frequently used vasopressor (*n* = 66, 41.8%).

ICU mortality was 60.7% (*n* = 97/160). Four additional patients died in the hospital. Seventy patients (43.8%) developed complications. From these, sepsis (*n* = 48, 30%) was the most common followed by re-intubation (*n* = 27, 16.9%) and hospital-acquired pressure injury (*n* = 18, 11.3%) (Table 2).

**Figure 2:**
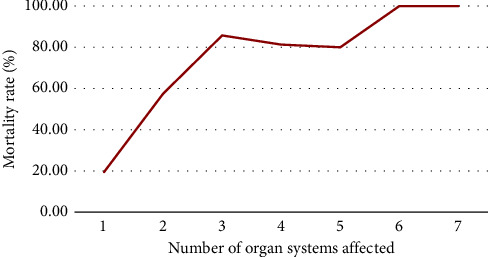
A line graph showing the number of organ systems affected in relation to their mortality rate, among mechanically ventilated patients admitted to the adult intensive care unit of Tikur Anbessa Hospital, from September 2019 to September 2020, Addis Ababa, Ethiopia.

As the number of organ systems affected increased, the probability of death also increased (Figure 2). On multivariable analysis, a low Glasgow Coma Scale (<8/7T) increased the odds of mortality by six times. Participants with a cardiovascular admission diagnosis were 5 times more likely to die (AOR 5.05, 95% CI 1.80–14.15). In addition, very low serum albumin (<2 g/dl) increased the odds of mortality by 4.9 times (Table 3).

## 4. Discussion

Mechanically ventilated patients have a very high mortality rate in our ICU, which is comparable with data from other low- and middle-income countries [9, 13, 18]. A low Glasgow Coma Scale, a cardiovascular diagnosis, and a very low serum albumin level were independent predictors of death in multivariable logistic regression.

Several findings from our study are worth noting. First, similar to previous studies from Ethiopia [1, 5, 19] and Kenya [13], patients with cardiovascular illness had a high mortality rate in this study. This finding may be due to the underlying disease condition coupled with difficulty in managing heart-lung interactions during mechanical ventilation [7]. In addition, there are no respiratory therapists in the medical and surgical ICUs. Mechanical ventilators are managed by clinicians with limited training [18], potentially affecting patient outcomes. Second, similar to other studies [5, 13, 18], we found that patients with low GCS were more likely to die. The change in mental status may represent an indicator of severe illness or a primary neurological disease. Third, a shorter hospital stay before ICU admission was associated with a worse outcome in univariable analysis, in contrast to other work [10]. This observation suggests that nonsurviving patients likely received care elsewhere and were referred to our hospital with a higher severity of illness. Fourth, very low serum albumin (when measured) was associated with higher mortality, as shown previously [20]. Our patient population is mostly malnourished due to poor socioeconomic status and then exposed to a catabolic state of illness without access to nutritionists or standardized nutritional therapy. Enteral feeding is not directed to the patients' calorie requirements and parenteral nutrition is unavailable.

Overall, the high mortality rate raises questions about the effective utilization of available resources. Half the patients (48.8%) were admitted with an ICU priority III or IV diagnosis, with a reduced chance of benefiting from ICU admission. This point may highlight an opportunity to better utilize limited resources.

This study has several strengths, as it is one of relatively few studies conducted in low- and middle-income countries of risk factors for mortality among mechanically ventilated patients. The results of this study can be used as a baseline and reveal several areas for improvement for different stakeholders. Nonetheless, it was limited by its single center and retrospective nature and lack of inclusion of all factors of potential interest, such as nutritional status and ventilatory parameters. Residual confounding and the impact of missing data limt the certainty of associations found. Multicentre observational studies using routinely and reliably collected data, or conducted prospectively, are required to advance the understanding of critical illness in Ethiopia [21–25].

## 5. Conclusions

ICU mortality in mechanically ventilated patients in this single hospital in Ethiopia is high. Coma, a cardiovascular diagnosis, and a very low serum albumin level are associated with an increased risk of death. The most commonly identified complication was infection. A multifaceted approach focused on developing skilled health human resources and context-appropriate guidelines on admission, nutrition, ventilator management, and infection prevention may prove effective in reducing mortality.

## Figures and Tables

**Table 1:** Baseline characteristics of mechanically ventilated patients admitted to the adult intensive care unit of Tikur Anbessa Hospital, Addis Ababa, Ethiopia from September 2019 to September 2020, displayed against their ICU outcome using the chi-square (X^2^), *t*-test, and Mann–Whitney *U* test.

Variables		Total (*n* = 160)	Survivors (*n* = 63)	Nonsurvivors (*n* = 97)	*P* value
Sex	Male Female	75 (46.9) 85 (53.1)	28 (44.4) 35 (56.6)	47 (48.5) 50 (51.5)	0.66
Age years	(Mean ± SD)	38.9 ± 16.2	36.4 ± 14.4 yrs	40.5 ± 17.2 yrs	0.123
Source of admission	OR Emergency Wards	70 (43.8) 38 (23.8) 48 (30.0)	39 (61.9) 9 (14.3) 13 (20.6)	31 (32.0) 29 (29.9) 35 (36.1)	<0.01
Hospital stay before admission, days	Median (IQR)	6 (2–14)	10 (5–15)	4 (1–12)	0.01
Place of admission	MICU SICU	66 (41.3) 94 (58.7)	10 (18.9) 53 (81.1)	56 (57.7) 41 (42.3)	<0.01
MPM II score	(Mean ± SD)	39.6 ± 25.5	21.9 ± 14.6	46.5 ± 26.4	<0.01
ICU priority level	Level 1 Level 2 Level 3 Level 4	77 (48.1) 5 (3.1) 47 (29.4) 31 (19.4)	52 (82.5) 2 (3.2) 8 (12.7) 1 (1.6)	25 (25.8) 3 (3.1) 39 (40.7 30 (30.9)	<0.01
GCS at ICU admission	<8/7T >8/8T/Sedated	35 (21.9) 125 (78.1)	3 (4.8) 60 (95.2)	32 (33.0) 65 (77.0)	<0.01
Indication	Operative Non-operative	68 (42.5) 92 (57.5)	41 (65.1) 22 (34.9)	27 (29.0) 70 (71.0)	<0.01
Admission diagnosis	Neurologic Respiratory Cardiovascular Hematologic Renal Gastrointestinal	85 (53.1) 97 (60.6) 57 (35.6) 45 (28.1) 41 (25.6) 25 (15.6)	45 (71.4) 25 (39.7) 7 (11.1) 8 (12.7) 8 (12.7) 6 (9.5)	40 (41.2) 72 (74.2) 50 (51.5) 37 (38.1) 33 (34.0) 19 (19.6)	<0.01
Initial mode of ventilation	AC/VC SIMV/VC AC/PC Others	87 (56.5) 41 (26.6) 15 (9.7) 11 (7.1)	23 (37.7) 26 (42.6) 4 (6.6) 8 (13.1)	64 (66.0) 15 (15.5) 11 (11.8) 3 (3.3)	<0.01
Serum albumin, g/dl	(Mean ± SD)	2.5 ± 0.8	3.0 ± 1	2.3 ± 0.6	<0.01
Comorbidity	Immunosuppression Hypertension Retroviral infection Congestive heart failure Diabetes	90 (56.3) 32 (20.1) 10 (6.3) 14 (8.8) 10 (6.3)	51 (52.6) 11 (17.5) 2 (3.2) 3 (4.8) 2 (3.2)	39 (61.9) 21 (21.9) 8 (8.2) 11 (11.3) 8 (8.2)	0.59

Process and outcome of care.

**Table 2:** Processes and outcome of ICU care of mechanically ventilated patients admitted to the adult intensive care unit of Tikur Anbessa Hospital, Addis Ababa, Ethiopia from September 2019 to September 2020, displayed against their ICU outcome using the chi-square (X^2^), *t*-test, and Mann–Whitney *U* test.

Process or outcome of care		Total (*n* = 160)	Survivors (*n *= 63)	Nonsurvivors (*n* = 97)	*P* value
Duration of ventilation, days	Median (IQR)	3 (2–7)	2 (2–5)	4 (2–8)	0.081
Blood transfusion		40 (25)	9 (22.5)	31 (77.5)	0.01
Vasopressor		75 (46.9)	2 (2.7)	73 (97.3)	<0.01
Hemodialysis		16 (10)	6 (37.5)	10 (63.5)	1.0
Complication in ICU	Sepsis Re-intubation Pressure injury	48 (30) 27 (16.8) 18 (11.3)	15 (23.8) 9 (14.3) 6 (9.5)	33 (34) 18 (18.6) 12 (12.4)	0.404
ICU length of stay	Median (IQR)	5 (2–10)	5 (3–10)	4.5 (2–11)	0.25
Total hospital stay, days	Mean (SD)	20.2 (17.9)	29.7 (16.9)	14.6 (16.0)	<0.01

**Table 3:** Predictors of mortality among mechanically ventilated patients admitted to the adult intensive care unit of Tikur Anbessa Hospital, from September 2019 to September 2020, Addis Ababa, Ethiopia.

Variable	COR (95%CI)	AOR (95%CI)	*P* value	*E*-value
Age	1.02 (0.99–1.04)	0.99 (0.97–1.02)	0.707	
GCS less than 8 or 7T	9.85 (2.87–33.83)	6.3 (1.19–33.00)	0.031^*∗∗∗*^	4.46
Cardiovascular diagnosis	8.51 (3.53–20.54)	5.05 (1.80–14.15)	0.002^*∗∗∗*^	3.92
Serum albumin less than 2 g/dl	14.79 (6.44–33.95)	4.9 (1.73–13.93)	0.003^*∗∗∗*^	3.85
MPM score	1.01 (1.03–1.08)	1.02 (0.99–1.05)	0.145	

^*∗∗∗*^ showing significant values. The model was able to classify 80.6% of the cases.

## Data Availability

The data used and/or analyzed for this study are available from the corresponding author upon reasonable request.

## Abbreviations

AOR: Adjusted odds ratio
CI: Confidence interval
COR: Crude odds ratio
CPR: Cardiopulmonary resuscitation
GCS: Glasgow Coma Scale
ICU: Intensive care unit
IQR: Interquartile/range
SD: Standard deviation
SPSS: Statistical Package for Social Sciences.
MPM: Mortality Prediction Model.
